# Isolation and phenotypic and genotypic characterization of the potential probiotic strains of *Lactobacillus* from the Iranian population

**DOI:** 10.1016/j.nmni.2021.100913

**Published:** 2021-07-06

**Authors:** H. Sedigh Ebrahim-Saraie, S. Khanjani, M. Hasannejad-Bibalan

**Affiliations:** 1) Razi Clinical Research Development Unit, Razi Hospital, Guilan University of Medical Sciences, Rasht, Iran; 2) Department of Microbiology, School of Medicine, Guilan University of Medical Sciences, Rasht, Iran

**Keywords:** Gut microbiome, Iran, *Lactobacillus*, lactic acid bacteria, probiotics

## Abstract

Among different causes of inflammatory bowel disease (IBD), the imbalance of the gut microbiome (dysbiosis) is one of the main reasons for the development of the disease. Probiotics are live microorganisms that can maintain gut microbiota by different mechanisms. We aimed to isolate and characterize the potential probiotic strains of *Lactobacillus* from the Iranian population. This cross-sectional study was conducted on faecal samples of 83 volunteer individuals living in Guilan Province, North Iran. The primary identification of *Lactobacillus* strains was performed by standard microbiological tests and confirmed by amplification of 16s rRNA specific primers. The acid and bile salt tolerance were assessed for all recovered strains. Also, the presence of 3 bacteriocins encoding genes was investigated by the PCR method. Totally, 42 samples were positive for *Lactobacillus* species. Acid and bile resistance assay showed that 67% and 33% of strains were resistant to acid and bile salt stress, respectively. Therefore, we found out that 28% of our *Lactobacillus* strains have the ability for resistance to acid and bile conditions. PCR results revealed that the prevalence of gassericin A, plantaricin S, lactacin bacteriocin genes were 16.6%, 12%, and 9.5%, respectively. Meanwhile, 5 out of 12 *Lactobacillus* strains that were resistant to acid and bile conditions contained one of the gassericin or plantaricin bacteriocins. We isolated 42 potential probiotic strains of *Lactobacillus*, of which the results of 5 strains were more promising and can be considered as potential probiotics sources for future functional products.

## Introduction

Inflammatory bowel disease (IBD) is a chronic inflammatory disease that involves the gastrointestinal tract [1]. Among different causes of IBD, the imbalance of the gut microbiome (dysbiosis) is one of the main reasons for the development of the disease [2,3]. The epidemiological studies suggest that the global prevalence of IBD is increasing and has become a significant public health challenge [[4], [5], [6]]. The gut microbiome consists of various microorganisms that gradually change based on the host and diet factors, but lactic acid bacteria (LAB) usually have a vital role in improving intestinal microbial balance [[7], [8], [9]].

Probiotics are live microorganisms that can maintain gut microbiota and affecting its composition and activity by different mechanisms [10]. *Lactobacillus* and *Bifidobacterium* are widely used strains in the production of probiotics and proved to have beneficial effects on host health [10,11]. LAB can improve host health by preventing pathogenic invasion by producing antimicrobial peptides (AMPs), modulate the microenvironment by producing lactic and acetic acids, and regulate the host immune system and cytokine profile [[12], [13], [14]]. LAB must show some properties to select as probiotic bacteria include tolerance of gastric acid, resistance to bile salts and bacteriocin profile [12,15]. The ability of LAB to resist acid and bile are two important factors that could indicate the potential of these strains to maintain their health benefits during transmission through the stomach and intestine [[16], [17], [18]].

Bacteriocins are heterogeneous peptides with potent antibacterial activity against pathogenic bacteria that are used as natural food preservation in many countries [19]. Modulate the host immune system, competition with pathogens for attachment to intestinal epithelial cells and improves gastrointestinal function are the main functions attributed to bacteriocins [[20], [21], [22]].

The probiotic characterization of LAB is different in the population of countries, more likely because of different genetic and environmental contributors [23]. Therefore, we aimed to isolate and characterize the potential probiotic strains of LAB from the Iranian population.

## Materials and methods

### Study design and sampling

This cross-sectional study was conducted during six months period in 2019 on faecal samples of 83 volunteer individuals that living in Rudbar city in the north of Iran. Rudbar is a non-industrial city with a humid and rainy climate, and vegetables are a major part of the diet of people in this area. Samples were taken from volunteer’s who were referred to rural health centres by convenience sampling method. Volunteer’s selection was according to two main criteria, lack of antibiotics consumption and any gastrointestinal diseases over six months period before sample collection. This study was approved by the Ethics Committee of Guilan University of Medical Sciences (IR.GUMS.REC.1398.016) and is in compliance with the declaration of Helsinki.

### Phenotypic and molecular identification

All samples were transferred to the microbiology laboratory, and after preparation of serial dilutions in PBS (pH = 7.4), 100 ml of each sample was cultured on Man, Rogosa and Sharpe (MRS) agar medium (Merck, Germany) and incubated anaerobically at 37 °C for 24 h. Then the pure colonies of each plate (∼10 colonies from each sample) was identified based on standard microbiological tests. All phenotypically confirmed isolates were identified by amplification of *Lactobacillus* 16s rRNA specific primers for-*lac* (5′-TGGAAACAGGTGCTAATACCG-3′) and Rev-*lac* (5′-CCATTGTGGAAGATTC CC-3′) [24].

### Acid tolerance test

The acid tolerance assay was performed according to Gopal et al. and Shehata et al. studies [25,26]. Briefly, 1 ml of MRS broth fresh culture comprising 10^9^ CFU/ml of *Lactobacillus* strains was added into 9 ml modified PBS with pH 3 and inoculated at 37 °C for 3 h. After incubation time, a serial dilution of each sample was made using sterile PBS, and the number of viable *Lactobacillus* strains was determined by plate colony count using MRS agar. The survival rate of *Lactobacillus* strains was measured by counting the cells. *Lactobacillus acidophilus* ATCC 1098 was used as control strains.

### Bile tolerance test

The bile salt tolerance assay was conducted as described before by Gopal et al. and Shehata et al. studies [25,26]. In brief, the *Lactobacillus* strains were cultured in 9 ml of fresh MRS broth with and without 0.4% (w/v) oxgall bile (Sigma) and incubated at 37 °C for 6 h. After incubation time, the growth rate was measured at 600 nm by using a spectrophotometer, and the coefficient of inhibition (Cinh) was calculated. *L. acidophilus* ATCC 1098 were used as control strains.

### DNA extraction and bacteriocin detection

DNA extraction of strains was done by using the Genomic DNA mini kit (Roche, Germany) based on the kit procedure. The presence of genes encoding three bacteriocins included gassericin A (*gaaA*), plantaricin S (*plnS*) and lactacin (*laf*) were determined by PCR assay with specific primers (Table 1) [[27], [28], [29]]. Identification of potentially probiotic *Lactobacillus* species was performed by PCR using species-specific primers described by Kwon et al. previously [30].

**Table 1:** List of used primers in the present study

Genes	Primers	Sequences (5ʹ→3ʹ)	Size (bp)	Ref
*gaaA*	Forward	GAACAGGTGCACTAATCGGT	800	[9]
	Reverse	CAGCTAAGTTAGAAGGGGCT		
*plnS*	F	GCCTTACCAGCGTAATGCCC	320	[10]
	R	CTGGTGATGCAATCGTTAGTTT		
*laf*	F	AGTCGTTGTTGGTGGAAGAAAT	184	[11]
	R	TCTTATCTTGCCAAAACCACCT		

### Statistical analysis

Results analysis was performed by using SPSS™ software, version 21.0 (IBM Corp., USA). The results are presented as descriptive statistics in terms of relative frequency. Values were expressed as the mean ± standard deviation (continuous variables) or percentages of the group (categorical variables).

## Results

Of a total of 83 faecal samples that were collected from volunteer individuals, 42 samples were positive for *Lactobacillus* species by standard microbiological tests and molecular confirmation. Of 42 samples, 27 (64%) were obtained from females and 15 (36%) from male individuals. The mean age of the participants was 20.6 ± 10.8 (Mean ± SD) years, and the age range was from 3 to 40 years. Acid and bile resistance assay showed that of 42 *Lactobacillus* strains, 28 (67%) strains were resistant to acid and survived in pH 3. Moreover, after 6 h of exposure to 0.4% (w/v) oxgall, 14 (33%) strains showed bile resistance. Also, 12 isolates (28%) have the ability for resistance to acid and bile conditions (Table 2). The mean age of the participants, which these 12 strains recovered from them, was 19.9 ± 8.9 years. Molecular detection revealed that *Lactobacillus plantarum* (66.7%) were the most prevalent species followed by, *Lactobacillus gasseri* (25%), and *Lactobacillus reuteri* (8.3%).

**Table 2:** The detailed characterization of 12 *Lactobacillus* strains that are simultaneously resistant to low pH and bile salt

No.	Strain	Sample source	Acid resistance (log CFU/ml)		Bile resistance (0.4%, h_6_)	Gene pattern
		Male or Female/Age (year)	Initial (h_0_)	Final (h_3_)	Coefficient of inhibition	
1	*L. gasseri*	M/22	8.89 ± 0.11	7.85 ± 0.15	0.32	*gaaA*
2	*L. plantarum*	F/34	9.38 ± 0.04	9.65 ± 0.06	0.29	No gene
3	*L. plantarum*	F/15	9.22 ± 0.19	8.71 ± 0.28	0.22	No gene
4	*L. plantarum*	F/3	9.68 ± 0.12	8.35 ± 0.42	0.36	*plnS*
5	*L. plantarum*	F/18	9.57 ± 0.88	9.91 ± 0.39	0.25	No gene
6	*L. plantarum*	F/8	8.69 ± 0.17	7.29 ± 0.09	0.09	*plnS*
7	*L. plantarum*	F/31	9.36 ± 0.12	9.89 ± 0.19	0.02	No gene
8	*L. reuteri*	M/16	8.74 ± 0.41	8.58 ± 0.46	0.19	No gene
9	*L. gasseri*	F/24	9.66 ± 0.42	9.26 ± 0.08	0.26	*gaaA*
10	*L. gasseri*	M/27	9.59 ± 0.11	9.89 ± 0.04	0.37	No gene
11	*L. plantarum*	F/19	9.87 ± 0.08	8.62 ± 0.74	0.27	*plnS*
12	*L. plantarum*	F/22	8.37 ± 0.05	8.52 ± 0.06	0.26	No gene

PCR results of 42 *Lactobacillus* strains revealed that the prevalence of bacteriocin genes were 7 (16.6%) *plnS*, 5 (12%) *gaaA*, and 4 (9.5%) *laf*. Also, the three strains that contained *plnS* gene and the two strains that contained *gaaA* gene were resistant to acid and bile. Totally, 5 out of 12 *Lactobacillus* strains that were resistant to both low pH and bile salt stress contained one of the gassericin or plantaricin bacteriocins. The mean age of the participants, which these five strains recovered from them, was 15.2 ± 9.2 years.

## Discussion

The gut microbiome plays an essential role in human health and disease progression [31].

*Lactobacillus* species, as the most widely used probiotics have more potential beneficial effects than other LAB [32,33]. Adaptation to the human gastrointestinal tract, such as bile and acid tolerance and the ability of bacteriocin production of *Lactobacillus* strains, are major criteria for considering them as a probiotic [34,35].

In the current study, probiotic characterization of 42 native *Lactobacillus* strains isolated from the north of the country was evaluated. Totally, 67% of tested strains survived in the acidic condition that suggests that these strains have the potential ability to survive in the human stomach. Previously, closest to our findings, Kılıç et al. and Gu et al. [36,37] showed their *Lactobacillus* strains had a good survival rate in acidic conditions. However, some major limitations in both studies were seen, including restricting the study population to a particular age group and a lack of bacteriocin profile determination. In contrast, a study performed in Tehran [38], the capital of Iran, showed a lower rate of acid (26%) and bile tolerance (35%) among their recovered *Lactobacillus* strains compared to our strains, which can be due to diversity of diet composition of industrial cities and restricting the study population to infants.

In the present study, we used oxgall for bile assay because this substitute is similar to human bile. Overall, 33% of our *Lactobacillus* strains survived after 6 h of exposure to 0.4% oxgall that could be a good predictor of surviving in small intestine conditions. The bile resistance percentage that was reported from different studies was varied and unpredictable because bile concentration in studies was different, and the mechanism of tolerance is not understood [39]. Moreover, Kõll et al. [40] described that the potential probiotic effects of Lactobacillus strains, such as the ability of bile and acid tolerance, were strain-specific, which highlights the importance of testing several strains to find the best probiotic strains. Interestingly, we find out that 28% of our Lactobacillus strains have the ability for resistance to acid and bile conditions, which makes them a significant candidate for further investigation.

Lactobacilli could produce different metabolites such as organic acid, hydrogen peroxide, and bacteriocin that have an inhibitory effect against the microbial community, and bacteriocin have a key role in this process [20]. Molecular analysis of recovered isolates revealed that the frequency of gassericin, plantaricin and lactacin 16.6%, 12% and 9.5%, respectively. The low frequency of bacteriocins was also reported from different studies [27,28], because recent findings have shown that the bacteriocin genes are highly diverse and widely distributed among *Lactobacillus* strains.

As the main limitation of the present study, the lack of species determination for isolated *Lactobacillus*, and investigation of a wider range of bacteriocins can be mentioned.

In conclusion, in this study, we isolated 42 *Lactobacillus* strains from faecal samples of a healthy individual that showed potential probiotic properties. Of these, results of five strains that belonged to *L. plantarum*, and *L. gasseri* species were more promising and can be considered as potential probiotics sources for functional products. However, further investigations on the probiotic features of these strains are still required to reach a comprehensive conclusion.

## Funding

This study was supported by 10.13039/501100005421Guilan University of Medical Sciences, Grant No. 159.

## Transparency declaration

None to declare.

## Ethical declarations

This study was approved by the Ethics Committee of Guilan University of Medical Sciences (IR.GUMS.REC.1398.016) and is in compliance with the declaration of Helsinki.

## CRediT author statement

All authors contributed to data analysis, drafting or revising the article, gave final approval of the version to be published, and agree to be accountable for all aspects of the work.

## References

[1] Rohr M., Narasimhulu C.A., Sharma D., Doomra M., Riad A., Naser S. (2018). Inflammatory diseases of the gut. J Med Food.

[2] Le B., Yang S.H. (2018). Efficacy of Lactobacillus plantarum in prevention of inflammatory bowel disease. Toxicol Rep.

[3] DeGruttola A.K., Low D., Mizoguchi A., Mizoguchi E. (2016). Current understanding of dysbiosis in disease in human and animal models. Inflam Bowel Dise.

[4] Zuo T., Ng S.C. (2018). The gut microbiota in the pathogenesis and therapeutics of inflammatory bowel disease. Front Microbiol.

[5] Kaplan G.G., Windsor J.W. (2020). The four epidemiological stages in the global evolution of inflammatory bowel disease. Nat Rev Gastroenterol Hepatol.

[6] Jairath V., Feagan B.G. (2020). Global burden of inflammatory bowel disease. Lancet Gastroenterol Hepatol.

[7] Rinninella E., Raoul P., Cintoni M., Franceschi F., Miggiano G.A.D., Gasbarrini A. (2019). What is the healthy gut microbiota composition? a changing ecosystem across age, environment, diet, and diseases. Microorganisms.

[8] Krajmalnik-Brown R., Ilhan Z.E., Kang D.W., DiBaise J.K. (2012). Effects of gut microbes on nutrient absorption and energy regulation. Nutr Clin Pract.

[9] Zhu S., Jiang Y., Xu K., Cui M., Ye W., Zhao G. (2020). The progress of gut microbiome research related to brain disorders. J Neuroinflammat.

[10] Wieërs G., Belkhir L., Enaud R., Leclercq S., Philippart de Foy J.-M., Dequenne I. (2020). How probiotics affect the microbiota. Front Cell Infect Microbiol.

[11] Hemarajata P., Versalovic J. (2013). Effects of probiotics on gut microbiota: mechanisms of intestinal immunomodulation and neuromodulation. Therapeut Adv Gastroenterol.

[12] Vieco-Saiz N., Belguesmia Y., Raspoet R., Auclair E., Gancel F., Kempf I. (2019). Benefits and inputs from lactic acid bacteria and their bacteriocins as alternatives to antibiotic growth promoters during food-animal production. Front Microbiol.

[13] Lobionda S., Sittipo P., Kwon H.Y., Lee Y.K. (2019). The role of gut microbiota in intestinal inflammation with respect to diet and extrinsic stressors. Microorganisms.

[14] Mathipa M.G., Thantsha M.S. (2017). Probiotic engineering: towards development of robust probiotic strains with enhanced functional properties and for targeted control of enteric pathogens. Gut Pathogens.

[15] Reuben R.C., Roy P.C., Sarkar S.L., Alam R.-U., Jahid I.K. (2019). Isolation, characterization, and assessment of lactic acid bacteria toward their selection as poultry probiotics. BMC Microbiol.

[16] Kim M., Nam D.G., Kim S.B., Im P., Choe J.S., Choi A.J. (2018). Enhancement of viability, acid, and bile tolerance and accelerated stability in lyophilized Weissella cibaria JW 15 with protective agents. Food Sci Nutr.

[17] Kim M., Nam D.G., Kim S.B., Im P., Choe J.S., Choi A.J. (2018). Enhancement of viability, acid, and bile tolerance and accelerated stability in lyophilized Weissella cibaria JW 15 with protective agents. Food Sci Nutr.

[18] Horackova S., Vesela K., Klojdova I., Bercikova M., Plockova M. (2020). Bile salt hydrolase activity, growth characteristics and surface properties in Lactobacillus acidophilus.

[19] Meade E., Slattery M.A., Garvey M. (2020). Bacteriocins, potent antimicrobial peptides and the fight against multi drug resistant species: resistance is futile?. Antibiotics.

[20] Bibalan M.H., Eshaghi M., Rohani M., Pourshafie M.R., Talebi M. (2017). Determination of bacteriocin genes and antibacterial activity of lactobacillus strains isolated from fecal of healthy individuals. Int J Mol Cell Med.

[21] Monteagudo-Mera A., Rastall R.A., Gibson G.R., Charalampopoulos D., Chatzifragkou A. (2019). Adhesion mechanisms mediated by probiotics and prebiotics and their potential impact on human health. Appl Microbiol Biotechnol.

[22] Galdeano C.M., Cazorla S.I., Dumit J.M.L., Vélez E., Perdigón G. (2019). Beneficial effects of probiotic consumption on the immune system. Ann Nutr Metabol.

[23] Zommiti M., Feuilloley M.G., Connil N. (2020). Update of probiotics in human world: a nonstop source of benefactions till the end of time. Microorganisms.

[24] Rohani M., Noohi N., Talebi M., Katouli M., Pourshafie M.R. (2015). Highly heterogeneous probiotic Lactobacillus species in healthy iranians with low functional activities. PloS One.

[25] Gopal P.K., Prasad J., Smart J., Gill H.S. (2001). In vitro adherence properties of Lactobacillus rhamnosus DR20 and Bifidobacterium lactis DR10 strains and their antagonistic activity against an enterotoxigenic Escherichia coli. Int J Food Microbiol.

[26] Shehata M., El Sohaimy S., El-Sahn M.A., Youssef M. (2016). Screening of isolated potential probiotic lactic acid bacteria for cholesterol lowering property and bile salt hydrolase activity. Ann Agricul Sci.

[27] Stoyancheva G., Marzotto M., Dellaglio F., Torriani S. (2014). Bacteriocin production and gene sequencing analysis from vaginal Lactobacillus strains. Arch Microbiol.

[28] Macwana S.J., Muriana P.M. (2012). A ‘bacteriocin PCR array’for identification of bacteriocin-related structural genes in lactic acid bacteria. J Microbiol Method.

[29] Mohammadi F., Eshaghi M., Razavi S., Sarokhalil D.D., Talebi M., Pourshafie M.R. (2018). Characterization of bacteriocin production in Lactobacillus spp. isolated from mother's milk. Microbial Pathog.

[30] Kwon H.S., Yang E.H., Yeon S.W., Kang B.H., Kim T.Y. (2004). Rapid identification of probiotic Lactobacillus species by multiplex PCR using species-specific primers based on the region extending from 16S rRNA through 23S rRNA. FEMS Microbiol Lett.

[31] Ding R-x, Goh W.-R., Wu R-n, Yue X-q, Luo X., Khine W.W.T. (2019). Revisit gut microbiota and its impact on human health and disease. J Food Drug Anal.

[32] Azad M., Kalam A., Sarker M., Li T., Yin J. (2018). Probiotic species in the modulation of gut microbiota: an overview. Biomed Res Int.

[33] Teame T., Wang A., Xie M., Zhang Z., Yang Y., Ding Q. (2020). Paraprobiotics and postbiotics of probiotic Lactobacilli, their positive effects on the host and action mechanisms: a review. Front Nutr.

[34] Terpou A., Papadaki A., Lappa I.K., Kachrimanidou V., Bosnea L.A., Kopsahelis N. (2019). Probiotics in food systems: significance and emerging strategies towards improved viability and delivery of enhanced beneficial value. Nutrients.

[35] da Silva Sabo S., Mendes M.A., da Silva Araújo E., de Almeida Muradian L.B., Makiyama E.N., LeBlanc J.G. (2020). Bioprospecting of probiotics with antimicrobial activities against Salmonella Heidelberg and that produce B-complex vitamins as potential supplements in poultry nutrition. Sci Rep.

[36] Kılıç G.B., Karahan A.G. (2010). Identification of lactic acid bacteria isolated from the fecal samples of healthy humans and patients with dyspepsia, and determination of their pH, bile, and antibiotic tolerance properties. J Mol Microbiol Biotechnol.

[37] Gu R.-X., Yang Z.-Q., Li Z.-H., Chen S.-L., Luo Z.-L. (2008). Probiotic properties of lactic acid bacteria isolated from stool samples of longevous people in regions of Hotan, Xinjiang and Bama, Guangxi, China. Anaerobe.

[38] Davoodabadi A., Dallal M.M.S., Foroushani A.R., Douraghi M., Harati F.A. (2015). Antibacterial activity of Lactobacillus spp. isolated from the feces of healthy infants against enteropathogenic bacteria. Anaerobe.

[39] Knarreborg A., Jensen S.K., Engberg R.M. (2003). Pancreatic lipase activity as influenced by unconjugated bile acids and pH, measured in vitro and in vivo. J Nutr Biochem.

[40] Koll P., Mändar R., Marcotte H., Leibur E., Mikelsaar M., Hammarström L. (2008). Characterization of oral lactobacilli as potential probiotics for oral health. Oral Microbiol Immunol.

